# Microstructure and crystallographic texture data in modern giant clam shells (*Tridacna squamosa* and *Hippopus hippopus*)

**DOI:** 10.1016/j.dib.2023.109947

**Published:** 2023-12-14

**Authors:** Kimberley Mills, Duncan D. Muir, Anthony Oldroyd, Eleanor H. John, Nadia Santodomingo, Kenneth G. Johnson, Muhammad Ali Syed Hussein, Sindia Sosdian

**Affiliations:** a Cardiff University, School of Earth and Environmental Sciences, United Kingdom; b Natural History Museum, London, United Kingdom; c Borneo Marine Research Institute, Universiti Malaysia Sabah, Kota Kinabalu, Malaysia

**Keywords:** EBSD, SEM, Bivalve shell, Microstructure, Texture, Aragonite, Coral reefs

## Abstract

This article provides novel data on the microstructure and crystallographic texture of modern giant clam shells (*Tridacna squamosa* and *Hippopus hippopus*) from the Coral Triangle region of northeast Borneo. Giant clams have two aragonitic shell layers—the inner and outer shell layer. This dataset focuses on the inner shell layer as this is well preserved and not affected by diagenetic alteration. To prepare samples for analysis, shells were cut longitudinally at the axis of maximum growth and mounted onto thin sections. Data collection involved scanning electron microscopy (SEM) to determine microstructure and SEM based electron backscatter diffraction (EBSD) for quantitative measurement of crystallographic orientation and texture. Post-acquisition reanalysis of saved EBSD patterns to optimize data quality included changing the number of reflectors and band detection mode. We provide EBSD data as band contrast images and colour-coded orientation maps (inverse pole figure maps). Crystallographic co-orientation strength obtained with multiple of uniform density (MUD) values are derived from density distributed pole figures of indexed EBSD points. Raw EBSD data files are also given to ensure repeatability of the steps provided in this article and to allow extraction of further crystallographic properties for future researchers. Overall, this dataset provides 1. a better understanding of shell growth and biomineralization in giant clams and 2. important steps for optimizing data collection with EBSD analyses in biogenic carbonates.

Specifications Table

**Table:** 

Subject	Structural biology
Specific subject area	Microstructure and texture of marine carbonates
Data format	Raw: CRC, CPR, TIFF, M, txt Analyzed: XLSX
Type of data	Table (.XLSX) SEM images (.TIFF) EBSD band contrast images (.TIFF) EBSD grain orientation maps (.TIFF) Raw EBSD dataset files (.CRC, .CPR, .txt) Code for EBSD data analysis and plotting (.txt, .M)
Data collection	Sampling of giant clam shells (*Tridacna squamosa; Hippopus hippopus*) was carried out within the Coral Triangle region of northeast Borneo (Sabah, Malaysia) in April 2019. Microstructural arrangements of aragonite were identified from in-lens secondary electron images acquired with a scanning electron microscope (Zeiss Sigma HD field emission gun SEM). Crystallographic and textural characterization data were collected using electron backscatter diffraction (Nordlys-2 EBSD system). EBSD measured crystallographic preferred orientations (CPO) were indexed and refined post-acquisition using AZtec 6.0 software (Oxford Instruments). EBSD band contrast images, colour-coded orientation maps (inverse pole figure maps) and pole figures were processed in AZtec Crystal 2.2 (Oxford Instruments) and MTEX toolbox 5.7.0 for MATLAB software. Crystallographic co-orientation strength was extracted from pole figures and presented as multiple of uniform density (MUD) values.
Data source location	· Institution: Universiti Malaysia Sabah · City/Town/Region: Darvel Bay, East Sabah · Country: Malaysia · Samples: *Tridacna squamosa* (SS02BCT) (4° 51′ 57.2328′' N, 118° 11′ 34.8864′' E): Triangle reef, Darvel Bay. *Hippopus hippopus* (SS01BSN) (4° 58′ 57.684′' N, 118° 21′ 42.5268′' E): Sakar reef, Darvel Bay.
Data accessibility	SEM and EBSD images are highlighted in this article. Complete datasets are found at: Repository name: Mendeley Data Data identification number: 10.17632/2zfgjy27wg.5 Direct URL to data: https://data.mendeley.com/datasets/2zfgjy27wg/5

## 1 Value of the Data

• This dataset provides characterization of the microstructure and crystallographic texture of the shells of two species of giant clam (Tridacna squamosa; Hippopus hippopus). These data are necessary for understanding shell growth and biomineralization mechanisms in the fields of structural biology and (paleo)environmental reconstruction.
• These data provide an optimized method and guide for accurate and precise EBSD data collection in biogenic carbonates such as bivalves and corals. This method is applicable to samples with biomineral crystal sizes down to 1 µm.
• The dataset can benefit those who wish to improve and optimize EBSD data quality when determining crystallographic orientation in biogenic carbonates.
• Future researchers may use the raw EBSD data provided [1] to extract further textural and crystallographic properties of the giant clam shells. For example, the data may be used to investigate grain boundary misorientation and provide advanced characterization of material properties.

## 2 Background

Giant clams (Tridacninae) are iconic reef dwellers that fulfil critical ecological roles in tropical coral reef communities [2]. They also serve as ultra-high-resolution bioarchives to reconstruct past oceanographic conditions in tropical regions, where instrumental records are lacking [3]. Increased understanding of the microstructural and crystallographic architecture of giant clam shells is fundamental to provide information on biomineralization and skeletal organization in changing oceanic conditions [4]. In this dataset, one objective was to focus on the optimization of EBSD data quality for aragonitic giant clam shells by providing detailed information on EBSD data collection and post-processing steps. A second objective was to provide characterization of microstructure and crystallographic texture in two giant clam species frequently used for (paleo)environmental study (*T. squamosa; H. hippopus*).

## 3 Data Description

**Fig. 1:**
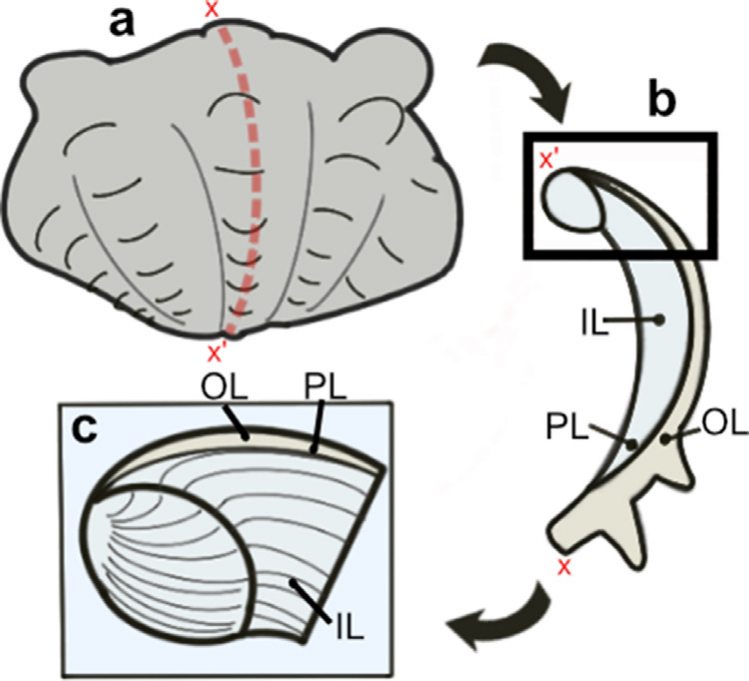
Schematic showing section location of a giant clam shell along the maximum growth axis. a) Shell valve with red vertical line indicating longitudinal cut along center. b) Longitudinal shell slice from umbo to upper shell margin highlighting the inner layer (IL), outer layer (OL) and pallial line (PL). Rectangle highlights region in c). c) Thin section 28×48 mm slide (60 µm thickness). Transect x-x’ shows how cut location in image a) relates to shell slice in b).

**Fig. 2:**
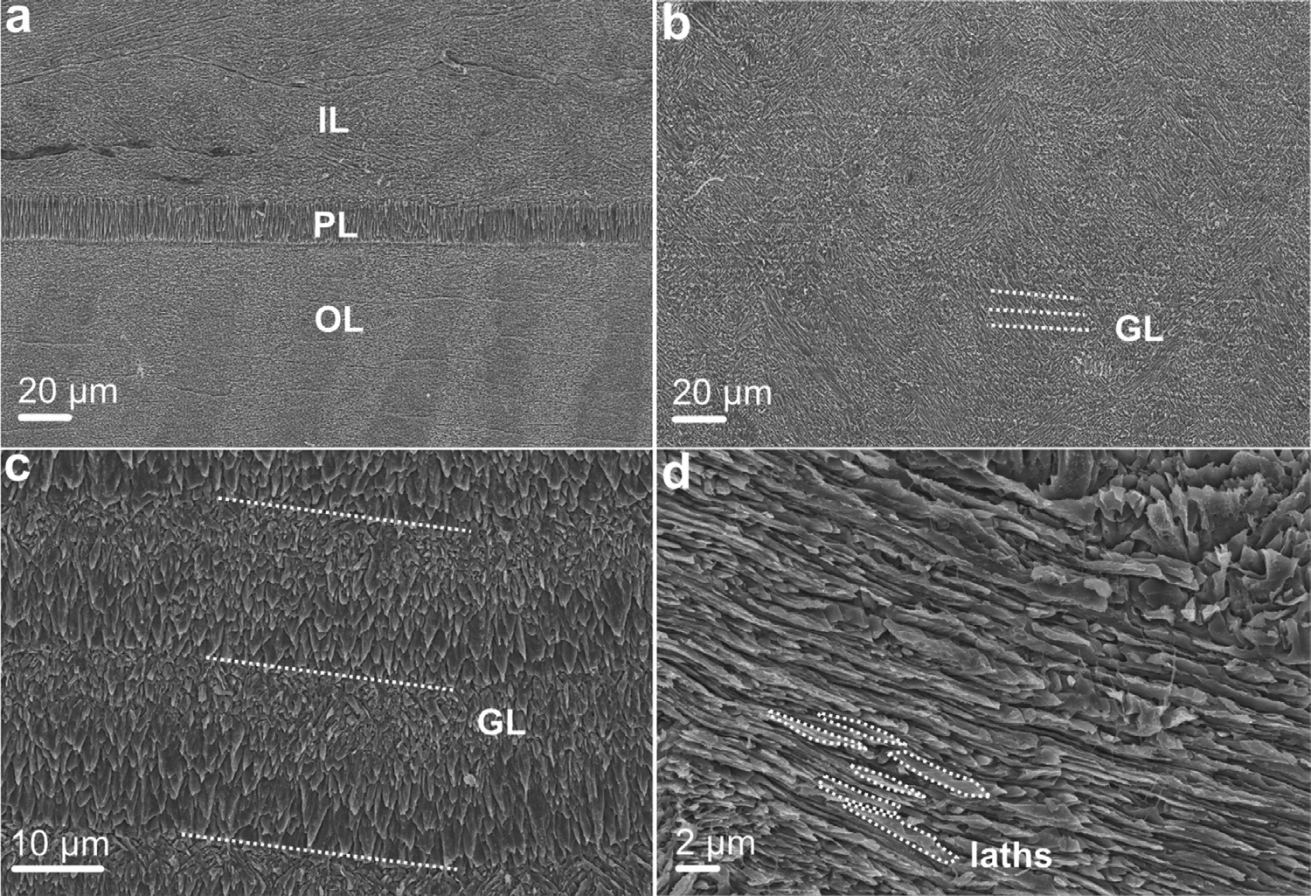
SEM in-lens secondary electron images showing the microstructure of Tridacninae shells. IL: inner layer; OL: outer layer; PL: pallial line; GL: growth lines. a) OL and IL divided by prismatic PL (specimen SS02BCT, *Tridacna squamosa*); b) daily GL in IL running perpendicular to direction of growth in complex crossed-lamellar microstructure (specimen SS02BCT, *Tridacna squamosa*); c) paired daily GL in IL that consist of a prismatic layer adjacent to smaller crystals (specimen SS01BSN, *Hippopus hippopus*); d) third order laths (biomineral units) that stack into larger layered structures (specimen SS02BCT, *Tridacna squamosa*).

**Fig. 3:**
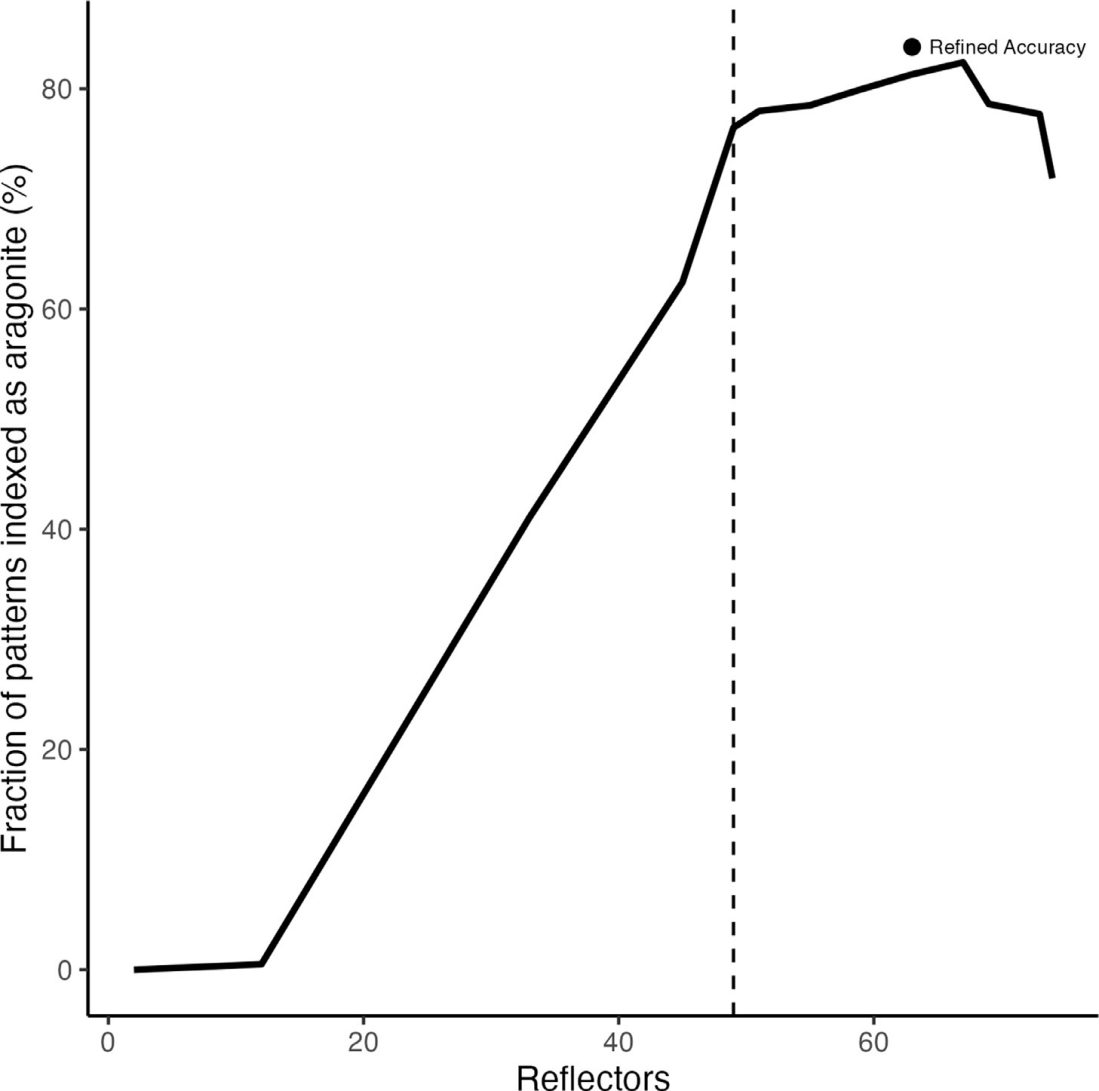
Relationship between fraction of indexed patterns as aragonite (%) and manual selection of number of reflectors for aragonite within the OINA database in AZtec 6.0 software (Oxford Instruments). Parameters used for the indexing of the aragonite unit cell were the OINA database (space group 62 Pmcn) *a* = 4.9614 Å, *b* = 7.9671 Å, *c* = 5.7404 Å. Single point is the refined accuracy band detection approach at 63 reflectors compared to routine Hough-based indexing. Dashed vertical line indicates the default number of reflectors (49) the software selects for aragonite.

**Fig. 4:**
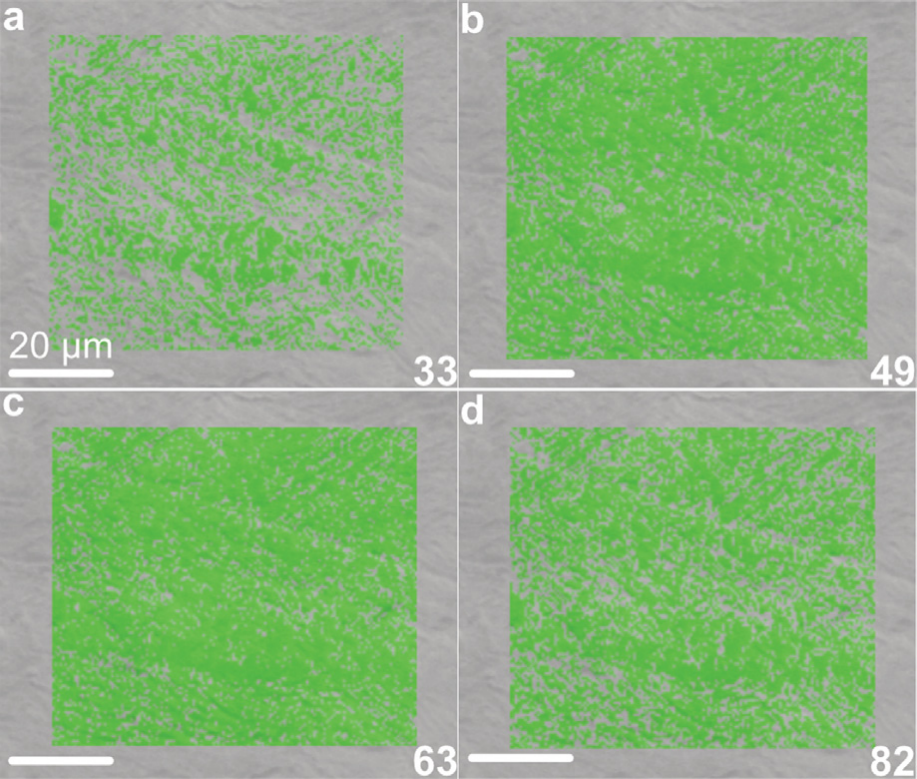
Fraction of indexed pattern for aragonite (%) by manually selecting the number of reflectors for aragonite with the OINA database in AZtec 6.0 software (Oxford Instruments). Patterns indexed had a mean angular deviation (MAD) below 1° a) 33 reflectors (41.06 % aragonite); b) 49 reflectors (76.48 % aragonite); c) 63 reflectors (81.31 % aragonite); d) 82 reflectors (64.66 % aragonite). Scale bars = 20 µm.

**Fig. 5:**
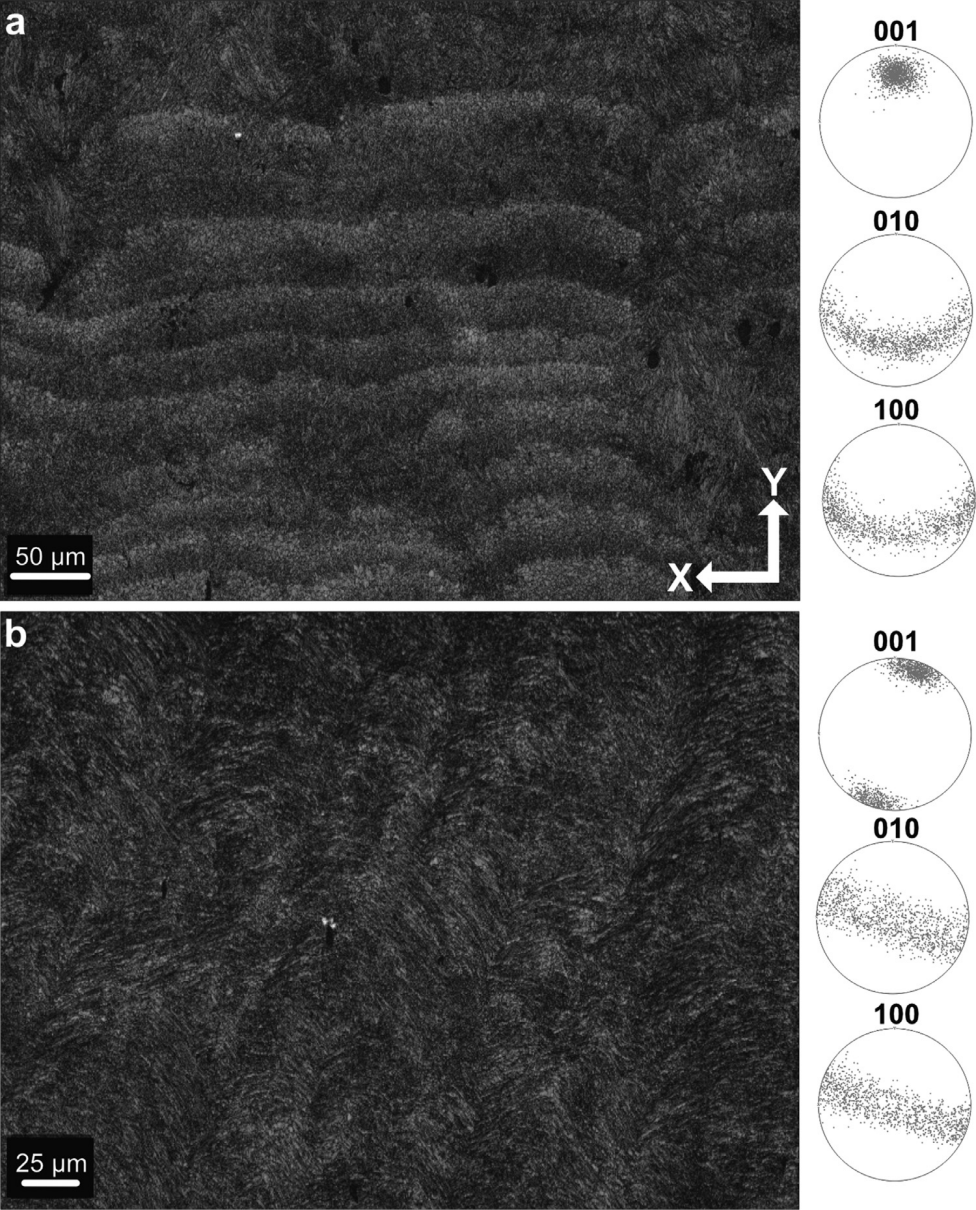
EBSD band contrast (pattern quality) images of the inner shell layer. a) Paired daily growth lines of *Hippopus hippopus* (SS01BSN) that correspond to IPF-Y map in Fig. 6 and the highlighted microstructure in Fig. 2c; c) complex crossed-lamellar microstructure of *Tridacna squamosa* (SS02BCT) that correspond to the IPF-Y map in Fig. 7 and highlighted microstructure in Fig. 2b. Associated pole figures display density distribution for image a) and b) respectively. Pole figures show indexed aragonite points with a preferred crystallographic orientation of the 001 axis approximately orthogonal to growth lines.

**Fig. 6:**
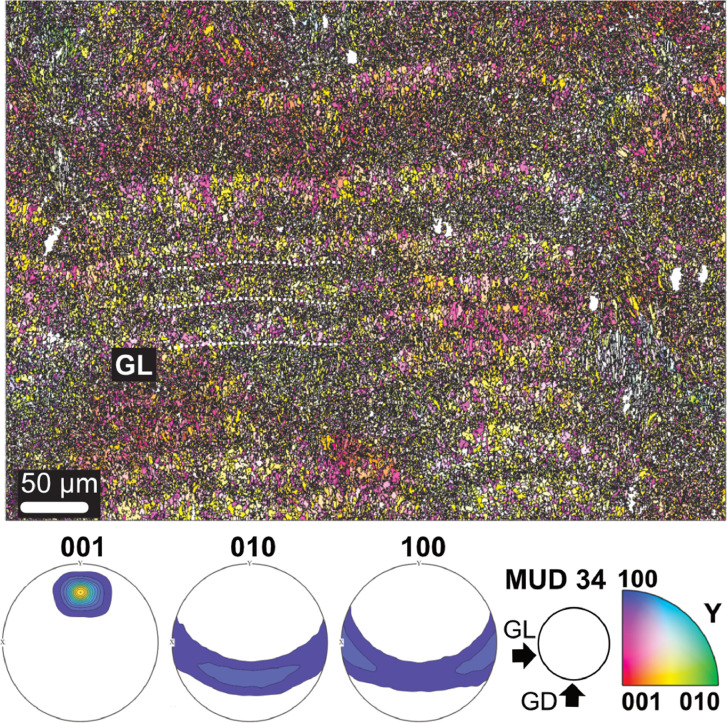
EBSD inverse pole figure (IPF-Y) map showing the microstructure and texture of paired daily growth lines within the inner layer of a *Hippopus hippopus* shell (SS01BSN), corresponding to band contrast image in Fig. 5a. Contoured pole figures show density distribution of all points indexed as aragonite and preferred crystallographic orientation of the 001 axis. GL: growth lines, GD: growth direction. Aragonite co-orientation strength has an MUD value of 34.

**Fig. 7:**
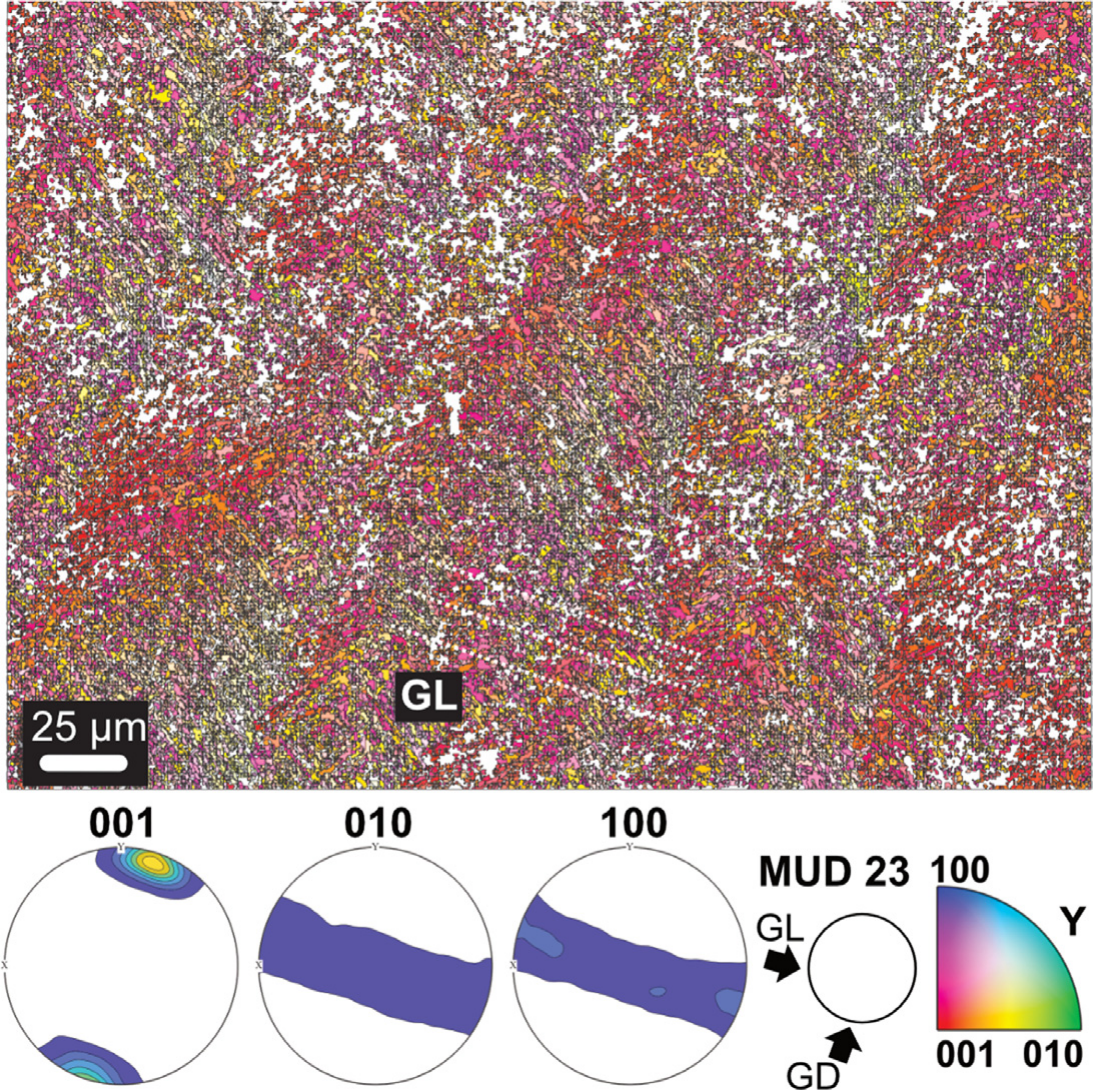
EBSD inverse pole figure (IPF-Y) map showing the complex crossed-lamellar microstructure and texture of the inner layer of a *Tridacna squamosa* shell (SS02BCT), corresponding to band contrast image in Fig. 5b. Contoured pole figures show density distribution of all points indexed as aragonite and preferred crystallographic orientation of the 001 axis. GL: growth lines, GD: growth direction. Aragonite co-orientation strength has an MUD value of 23.

Microstructure and crystallographic texture data for the shells of the giant clams *T. squamosa* and *H. hippopus* is presented herein. A schematic overview of sample preparation is given in Fig. 1—shell valves were sectioned longitudinally along the maximum growth axis, cut into ∼1–2 cm thick slices and mounted onto glass slides for preparation of thin sections. SEM in-lens secondary electron high-resolution images used for microstructural characterization of the material are presented as TIFF images. Fig. 2 focuses on the microstructure of the inner shell layer at a micro- to nanoscale, showing daily growth lines intersecting a complex crossed-lamellar microstructure (Fig. 2b) and paired daily growth lines that consist of a prismatic layer adjacent to smaller crystals (Fig 2c). Fig. 3, Fig. 4 show post-acquisition refinement of EBSD pattern indexing using varying numbers of reflectors (i.e. list of Kikuchi bands to be considered in the indexing process) and different band detection approaches (i.e. refined accuracy versus Hough-based band detection). EBSD band contrast images are presented with associated pole figures as TIFF images (Fig. 5), where dark pixels represent poor pattern quality and bright pixels represent high pattern quality. EBSD preferred crystallographic orientation (CPO) data are represented as color-coded orientation maps (TIFF images) and are shown with corresponding contoured pole figures in Figs. 6,7. Pole figures are a stereographic projection of aragonite planes with axes defined by an external reference frame (X, Y, Z correspond to E-W, N-S and out of plane respectively), showing clustering of points around specific direction(s) (i.e. pole maxima). The strength of the CPO is quantified using multiple of uniform distribution (MUD) values, which is derived from the maximum intensity of contoured pole figures (Figs. 6,7). Orientation maps are colored according to the inverse pole figure (IPF) color key for aragonite referenced to the Y direction of the external reference frame, where similar colors relate to similar orientations.

A tabulated version of post-acquisition indexing optimization is presented in Table 1, included in the Mendeley Data Repository as a .XLSX file. The repository also contains raw SEM EBSD files (.CRC, .CPR, .txt) for *T. squamosa* and *H. hippopus*, along with a sample script (.M, .txt) with code showcasing EBSD data analysis and plotting in MTEX toolbox 5.7.0 for MATLAB R2022b.

The files stored within the data repository are:

• SS02BCT.CRC: Raw EBSD dataset file for *Tridacna squamosa* (SS02BCT)
• SS01BSN.CRC: Raw EBSD dataset file for *Hippopus hippopus* (SS01BSN)
• SS02BCT.CPR: Raw EBSD dataset file for *Tridacna squamosa* (SS02BCT)
• SS01BSN.CPR: Raw EBSD dataset file for *Hippopus hippopus* (SS01BSN)
• SS02BCT.txt: Raw EBSD dataset file for *Tridacna squamosa* (SS02BCT)
• SS01BSN.txt: Raw EBSD dataset file for *Hippopus hippopus* (SS01BSN)
• EBSD_Tridacnidae.M: Code for EBSD data analysis and plotting.
• EBSD_Tridacnidae.txt: Code for EBSD data analysis and plotting.
• Reflectors.XLSX: Table of post-acquisition reanalysis of EBSD patterns stored at indexing using manual selection of reflectors.

## 4 Experimental Design, Materials and Methods

### 4.1 Sample preparation

Two modern giant clam shells, *T. squamos*a (fluted giant clam) and *H. hippopus* (bear paw clam) were collected from Darvel Bay (4° 51′ 57.2328″ N, 118° 11′ 34.8864″ E; 4° 58′ 57.684″ N, 118° 21′ 42.5268″ E respectively) within the Coral Triangle region of northeast Borneo (Sabah, Malaysia) in April 2019. The exterior of one valve of each shell was thoroughly rinsed and scrubbed to remove dirt and debris, before being air-dried. Afterward, valves were cut into ∼1–2 cm thick slices along the axis of maximum growth (longitudinal from umbo to upper shell margin) (Fig. 1a) with a HC Evans and Son (Eltham) LTD circular saw (250 mm blade, 1 mm thickness). Thin sections (∼60 µm thickness) cut perpendicular to the direction of growth were prepared from slices (Fig. 1b). One side of each cut slice was ground flat using silicon carbide 1000 grit. The slices were then washed, dried and stuck to 28×48 mm frosted glass slides using Araldite 2020 epoxy resin. Excess sample was cut from the slides leaving a 500–1000 µm slice stuck to the glass. Slides were then lapped on a Logitech LP50 lapper using 600 silicon carbide grit to leave samples at a thickness of 100 µm. Afterward, the slides were lapped by hand using 1000 silicon carbide grit until the required sample thickness had been reached. Slides were washed in an ultrasonic bath and samples polished on a Logitech PM5 lapper with 0.3 µm aluminum oxide. After polishing slides, they were again washed in an ultrasonic bath.

### 4.2 SEM

Polished sections of *T. squamosa* and *H. hippopus* were etched with 0.5% HCl for 15 s to improve visibility of biominerals and then rinsed for 1 min with deionized water. To dry samples, a canister of compressed air was sprayed gently across the surface of sections. Afterward, samples were sputter coated with a 20 nm thick layer of gold-palladium alloy (Au-Pd) using a BIO-RAD SC500 sputter coater at the School of Earth and Environmental Sciences, Cardiff University. A Zeiss Sigma HD field emission gun scanning electron microscope (FEG-SEM) at the School of Earth and Environmental Sciences, Cardiff University, was used under high vacuum for the characterization of aragonitic microstructures with focus on the inner shell layer of sections. The entire height of the inner layer of sections was examined with SEM for preliminary identification of microstructure across the whole surface (an area with height ∼30 mm, length ∼1 mm). The following SEM parameters were used to obtain in-lens secondary electron (SE) images of different microstructures (Fig. 2): 10 kV accelerating voltage, final aperture size 30 µm with a nominal beam current of 210 pA, working distance ∼9.5 mm, pixel dwell time 10 µs.

### 4.3 EBSD

Areas of thin sections selected for crystallographic and textural characterization with EBSD were based on prior microstructural identification with SEM. Sections were repolished and subjected to several sequential mechanical grinding and polishing steps, including a final polish with Logitech SF1 Polishing Suspension of colloidal silica using a Logitech PM5 automatic polisher (70 rpm rotation, 2 × 10 min cycles). Afterward, copper tape was applied in a rectangle around selected areas of samples for mapping to eliminate electron charging within the high-vacuum SEM chamber. Samples were coated with a thin uniform layer (3 nm) of carbon [5] using a Agar Turbo Carbon Coater. Fraction of the indexed pattern for aragonite (%) was tested with varying layers of carbon thickness between 2 and 6 nm, but 3 nm provided the strongest diffraction signal with negligible charging of the sample. EBSD mapping was carried out using a Zeiss Sigma HD FEG-SEM equipped with a Nordlys-2 EBSD detector at the School of Earth and Environmental Sciences, Cardiff University. In the SEM, samples were tilted at an angle of 70° at ∼10 mm working distance with ∼193–194 mm detector insert distance. Diffraction patterns were collected at a resolution of 0.5 µm step size, 20 kV accelerating voltage, 60 µm aperture in high current mode with a 2.7 nA nominal beam current and 2 × 2 camera (320×240 pixels) binning. Total acquisition time was 27 h for SS01BSN and 12 h for SS02BCT, with map dimensions of 1024×768 pixels and 681×510 pixels respectively. Exposure time was 96.8 ms for SS01BSN and 127.58 ms for SS02BCT.

Electron backscatter patterns were indexed using Oxford Instruments AZtec 6.0 software. Parameters chosen for the indexing of the aragonite unit cell were the OINA database *a* = 4.9614 Å, *b* = 7.9671 Å, *c* = 5.7404 Å space group 62 Pmcn [6]. Aragonite indexed with the OINA database provided pole figures with a preferred crystallographic orientation of the 001 axis orthogonal to growth lines.

### 4.4 EBSD post-acquisition refinement

Post-acquisition refinement to optimize index rates of aragonite was performed on data with EBSD patterns stored at indexing using AZtec 6.0 software (Oxford Instruments). Maps were reanalyzed changing the number of reflectors, band detection mode, Hough resolution and area of interest (AOI). Manual selection of the number of reflectors (i.e. list of Kikuchi bands to be considered in the indexing process) within the OINA and HKL databases ranged between 2 and 82 reflectors (Fig. 3). The relationship between fraction of indexed pattern for aragonite (%) and reflectors peaked at 67 reflectors in the OINA database, which increased indexing by 6 % compared to default selection of 49 reflectors while keeping mean angular deviation (MAD) under 1° (Fig. 3, Fig. 4; Table 1 in data repository). Refined accuracy band detection mode compared to routine Hough-based indexing further increased indexing by 1–3 %. Manual alteration of Hough resolution and area of interest (AOI) did not change the percentage of indexed pattern for aragonite.

### 4.5 EBSD data analysis

Data analysis was carried out in MTEX toolbox 5.7.0 for MATLAB R2022b [7]. Grains were reconstructed using a threshold angle of 2° Minimum grain size was set to 3 pixels in comparison to 10 pixels previously used for giant clam aragonite [8] because grain sizes were notably small (under 1 µm) in some areas. Points with mean angular deviation (MAD) over 1° were discarded and remaining grain boundaries smoothed. Zero solutions, that is missing data from parts of the sample that showed an absence of diffraction, were not interpolated to avoid over-simplification of the dataset in the presence of small grains. EBSD band contrast images, EBSD color-coded orientation maps (inverse pole figure maps) and pole figures for *T. squamosa* and *H. hippopus* were assembled using MTEX and are provided in Fig. 5, Fig. 6, Fig. 7. Pole figures were plotted on a lower hemisphere projection in the YX projection plane, with spread of the poles controlled by half-width [9]. An optimal half-width of approximately 4° for the data was computed based on the mean orientation of grains using the kernel function for orientation distribution function (ODF) estimation. Crystallographic co-orientation strength is presented as multiple of uniform density (MUD) values extracted from pole figures. The strength of the crystallographic preferred orientation is derived from the maximum intensity of contoured pole figures. MUD statistically measures sharpness of texture and a strong crystal co-orientation will have a higher MUD value than a low or random co-orientation (e.g. [9,10]).

## Limitations

The limitation of this dataset is that the EBSD data were generated from a singular specimen of each species investigated. This may hinder the reliable interpretation of MUD values to understand the variety of crystal co-orientation strength that exists between species. However, the primary aim of this study was to provide reproducible steps for future researchers, which we have laid out in this article. By providing these steps and raw EBSD data files, we suggest future researchers add more samples to further capture diversity in crystallographic features.

## Ethics Statement

The authors state that the present work meets the ethical requirements for publication in Data in Brief. Human or animal experiments were not conducted and social media data was not collected.

## CRediT authorship contribution statement

**Kimberley Mills:** Conceptualization, Methodology, Investigation, Visualization, Writing – original draft. **Duncan D. Muir:** Conceptualization, Methodology, Investigation, Writing – review & editing. **Anthony Oldroyd:** Resources, Writing – review & editing. **Eleanor H. John:** Conceptualization, Writing – review & editing. **Nadia Santodomingo:** Resources, Funding acquisition. **Kenneth G. Johnson:** Resources, Funding acquisition. **Muhammad Ali Syed Hussein:** Resources. **Sindia Sosdian:** Conceptualization, Writing – review & editing, Funding acquisition.

## Data Availability

Microstructure and crystallographic texture data from modern giant clam shells (Tridacna squamosa and Hippopus hippopus) (Original data) (Mendeley Data).
